# Comparative analysis of wastewater sample processing methods for antimicrobial resistance surveillance

**DOI:** 10.1128/spectrum.02089-25

**Published:** 2026-06-15

**Authors:** Helena Ferreira Leal, Arthur Ouradou, Nahla Koukene, Amina Barka, Caroline Quach, Émilie Bédard

**Affiliations:** 1 Department of Microbiology, Infectious Diseases and Immunology, Faculty of medicine, Université de Montréal (UdeM) 5622, Montréal, Quebec, Canada; 2 Department of Civil, Geological and Mining Engineering, Polytechnique Montréal 5596, Montréal, Quebec, Canada; 3 Centre Hospitalier Universitaire Sainte-Justine (CHU Sainte-Justine), Montréal, Quebec, Canada; University of Guelph College of Biological Science, Guelph, Canada

**Keywords:** antimicrobial resistance, antimicrobial resistance gene, wastewater treatment plant, wastewater-based epidemiology, DNA quantification, DNA extraction, microbial diversity, sample concentration, Qubit, nanodrop

## Abstract

Antimicrobial resistance monitoring in wastewater represents a key challenge, especially in light of the multiple methods available. Our study assessed the impact of selected sample processing techniques on microbial diversity and antimicrobial resistance gene recovery from wastewater. We analyzed 38 subsamples derived from 12 wastewater samples collected across three wastewater treatment plants over 4 months. We compared the effects of different centrifugation volumes (50, 250 mL), speeds (3,400 × *g* vs 15,000 × *g*), and pellet versus filter concentration methods, as well as the performance of two DNA extraction kits—DNeasy PowerSoil Pro Kit and DNeasy PowerLyzer Kit (Qiagen, DE)—on wastewater samples. Additionally, we compared the performance of two DNA quantification instruments, NanoDrop One and Invitrogen Qubit 4 (Thermo Scientific, US). The analysis involved 16S rRNA gene sequencing on an Illumina MiSeq platform (PE250, 10M reads) and quantitative PCR (qPCR) of 11 selected antimicrobial resistance genes (ARGs). Higher centrifugation speeds and larger sample volumes lead to improved DNA yield. Pellets generally exhibited higher (1.32 times) ARG recoveries compared to filters. DNA quantification methods showed strong positive correlation (Pearson R = 0.78, Spearman ρ = 0.81, *P* < 0.01) with each other and with qPCR data. The approach implemented in this study (centrifuging 200 mL samples at 15,000 × *g*, followed by extraction with the DNeasy PowerSoil Pro Kit) shows promise for antimicrobial resistance (AMR) monitoring.

**IMPORTANCE:**

To monitor the prevalence of antimicrobial resistance genes (ARGs) is crucial for anticipating and mitigating the spread of antimicrobial resistance (AMR). Urban wastewater provides community-level data to inform timely interventions. By establishing a method that balances efficiency and feasibility, this protocol represents a step toward a standardized wastewater surveillance (WS) framework for AMR, enhancing our ability to safeguard community health.

## INTRODUCTION

Wastewater surveillance (WS) has significantly evolved in recent years and is emerging as a pivotal public health tool (1–5). This approach has proven exceptionally valuable in tracking infectious diseases. Notably, the advancements of WS in detecting viral pathogens, including SARS-CoV-2, across various environmental matrices, such as wastewater, soil, and rainwater. The ability to timely monitor these diverse sources has catalyzed the establishment of global surveillance networks (6–8), demonstrating its capacity to provide real-time, community-level health data.

One of the critical applications of WS is monitoring antimicrobial resistance (AMR). Initiatives, such as the Global Sewage Surveillance project, have utilized WS to monitor antimicrobial resistance genes (ARGs) on a global scale, covering regions across Europe, Asia, Africa, and the Americas, enabling a comprehensive comparison of ARGs across diverse environmental and socio-economic settings (1, 2). Analyzing wastewater samples from human communities offers crucial insights into antimicrobial usage patterns and the emergence of ARGs, aiding the development of robust public health strategies against AMR (9–11).

However, technical challenges, particularly the lack of standardized sample processing protocols, currently limit the full potential of WS in this area (12, 13). The diversity of technical parameters used in various studies hinders the comparability and interpretation of data across different settings. Additionally, the lack of standardization in sampling methods, including variations in sampling volume and frequency, further complicates cross-study comparisons and impacts the reliability of the data (13).

Although wastewater samples are generally concentrated, an additional concentration step is often necessary to enhance the recovery of low-abundance organisms or genetic material, ensuring sufficient yield for downstream analysis. Standardizing the volume of concentrated wastewater is a crucial aspect of protocol optimization (14, 15).

The volume selected significantly influences the representativeness of the microbial community, affecting the detection and relative abundance of specific targets, such as ARGs (16, 17). Smaller sample volumes or samples collected closer to specific sources may not represent the broader community’s microbial diversity adequately, potentially leading to an overrepresentation of bacteria from a limited number of individuals (3, 18). On the other hand, larger volumes are likely to include more diverse bacterial populations, providing a more comprehensive view of the microbial community composition. However, processing large volumes requires thorough homogenization due to the potential presence of larger particles, which, in settings with a small contributing population and limited wastewater mixing—such as hospital effluents—may increase the likelihood of over-representing bacteria from a few individuals rather than capturing a fully homogenized microbial community (17, 19).

Centrifugation speed is another critical factor requiring careful consideration during protocol development. This process substantially affects the efficiency of bacterial component separation and the integrity of the recovered DNA (20). High-speed centrifugation at a rate exceeding 5,000 × *g* can cause cell surface damage and reduce the viability of certain bacterial strains, thus altering the microbial community composition (20–22). This is an important consideration for culture-dependent methods. In contrast, higher centrifugation speeds might be more suitable in DNA-based diversity studies, where the focus is on DNA rather than viable bacteria. This is particularly relevant when processing wastewater samples, where lower speeds might result in incomplete separation of particulate matter (17). However, it is essential to carefully select the appropriate centrifugation conditions, as excessively high speeds can still damage DNA through shear forces, particularly when it is in a free solution (23).

The decision to use pellets or filters for DNA extraction is critical, as it can influence both the quantity and representativeness of the extracted genetic material. Pellets, formed through centrifugation, are likely to yield greater biomass, potentially leading to increased DNA recovery (15, 20). Conversely, filters, typically used for capturing bacteria from larger volumes, may provide a more comprehensive representation of the microbial community (24, 25).

Post-DNA recovery, quantifying and assessing its quality is crucial for downstream techniques, such as high-throughput sequencing and quantitative PCR (qPCR). While both spectrophotometric and fluorometric methods are widely used (26, 27), they have unique characteristics that necessitate thorough evaluation to determine their suitability for specific analytical needs.

Fluorometric methods, particularly those using intercalating dyes, such as Invitrogen Qubit 4, reportedly excel in sensitivity, precisely measuring DNA concentration (28–30). Moreover, due to the reagents binding selectively to the nucleic acid of interest, Invitrogen Qubit 4’s specificity ensures accurate concentration readings in the presence of contaminants.

In contrast, spectrophotometry assays, such as NanoDrop One, operate on ultraviolet absorbance, providing concentration measurements and purity analysis through 260/280 and 260/230 ratios, which indicate contamination levels from proteins and salts (26, 27, 29, 30). Its ease of use, requiring no reagent preparation, is a significant advantage. Nevertheless, this method is less sensitive than Invitrogen Qubit 4, with reduced accuracy for samples with a concentration less than 10 ng/mL (31).

In our investigation, we explored key parameters of sampling concentration protocols that contribute to high DNA recovery, ensure taxonomic representativeness, and maintain DNA quality suitable for downstream applications. We evaluated the performance of two extraction kits, DNeasy PowerSoil Pro and DNeasy PowerLyzer PowerSoil (Qiagen, DE), and the differences between two widely used DNA quantification methods, NanoDrop One and Invitrogen Qubit 4 (Thermo Scientific, US). Aiming for methodological refinement and technical precision, this research provides a descriptive and comparative assessment of WS approaches relevant to AMR monitoring.

## MATERIALS AND METHODS

This study examined a wastewater sample processing protocol initially conceived for SARS-CoV-2 detection by N’Guessan et al. (4), adapting it for AMR surveillance.

### Testing conditions

**Fig 1:**
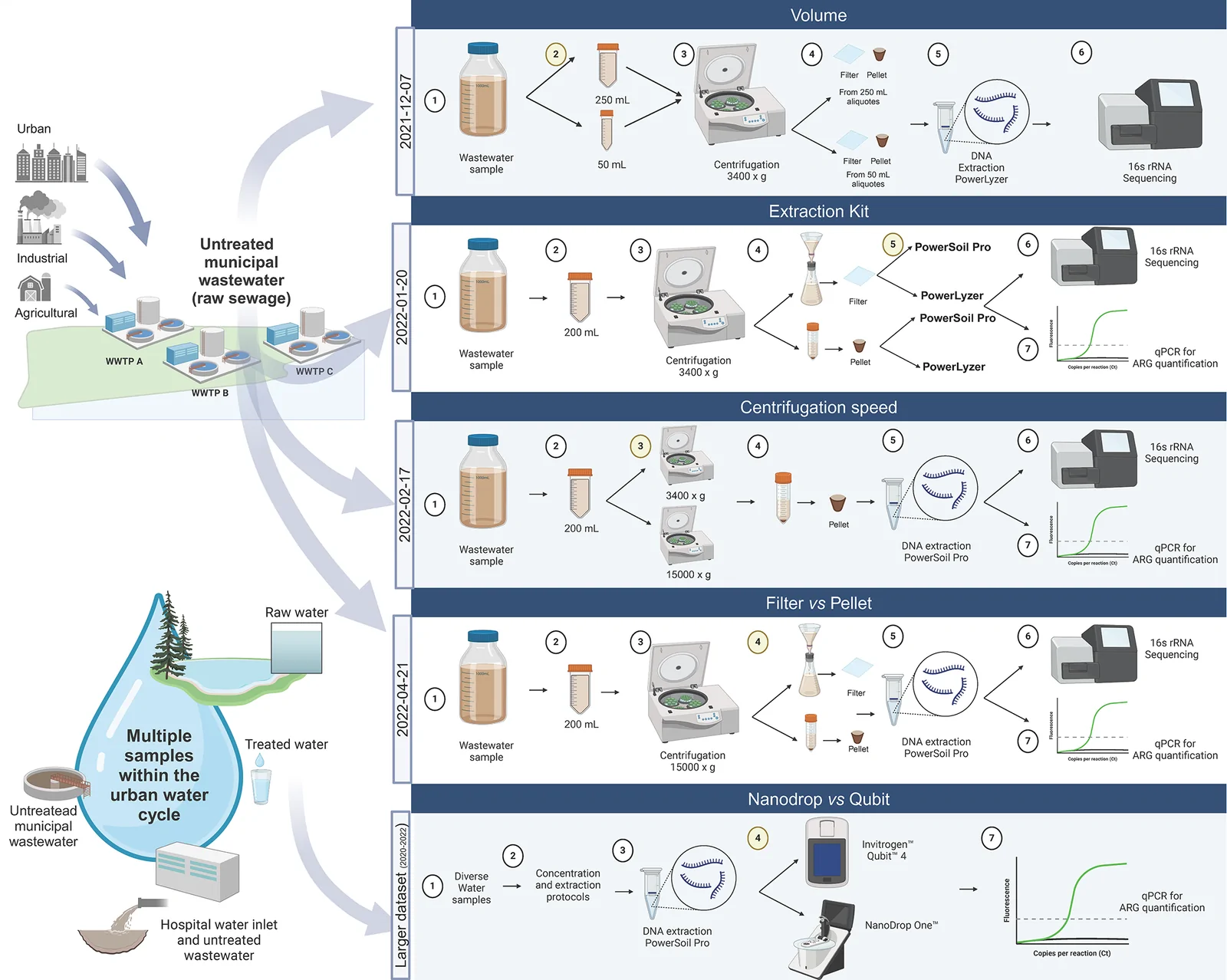
Overview of the experimental workflow and methodological comparisons across wastewater samples. The diagram summarizes the experimental design used to evaluate different concentrations and extraction protocols for wastewater-based AMR monitoring. On the left, untreated municipal wastewater samples from three urban treatment plants (WWTP A, B, and C) are collected at four time points (December 2021 to April 2022), along with additional samples from the broader urban water cycle (bottom). On the right, each experimental block illustrates one of the five methodological comparisons performed: (1) volume: two concentration volumes (250 and 50 mL) were compared for both pellet- and filter-based approaches; (2) extraction kit: DNA was extracted using either the DNeasy PowerLyzer or PowerSoil Pro kits; (3) centrifugation speed: pellets were recovered at either 3,400 × *g* or 15,000 × *g*; (4) filter versus pellet: comparison of biomass recovery strategies following filtration or centrifugation; and (5) DNA quantification methods: comparison between fluorometric (Invitrogen Qubit 4) and spectrophotometric (NanoDrop One) DNA measurements. The protocols were followed by DNA extraction and analysis using 16S rRNA gene sequencing for microbial diversity and qPCR for quantification of ARGs. Arrows and numbers indicate the stepwise sequence of procedures within each workflow.

Different sampling processing conditions were assessed (Fig. 1). Untreated wastewater samples (influent) were collected from December 2021 to April 2022 at three wastewater treatment plants (WWTPs) located in a major urban area of Quebec, Canada. For each WWTP—designated A, B, and C—autosamplers were used to collect 1-L composite samples over 24 h.

The samples were transported in a cooler with ice packs to maintain a temperature of 4°C during transit to the laboratory (30–40 min). Upon arrival, the samples were immediately homogenized and processed. The pellets and filters were stored at −20°C before DNA extraction.

Samples were subjected to various processing conditions, including different volumes, centrifugation speeds, concentration methods, and DNA extraction kits, to evaluate their impact on microbial diversity and ARG prevalence. Total suspended solids (TSS) and volatile suspended solids (VSS) were measured following the Standard Methods for the Examination of Water and Wastewater (32) and are expressed in mg/L. The sample descriptions, testing conditions, and TSS and VSS values are detailed in Table 1.

**TABLE 1:** Samples and processing conditions used in preliminary tests

Collection date	WWTP	Sample ID	TSS (mg/L)	VSS (mg/L)	Concentrated volume (mL)	Concentration type	Centrifugation speed (× *g*)	Extraction kit
	**Volume**							
12-07-2021	A	34	324	252	50	Pellet	3,400	PowerLyzer
		35			250	Pellet		
		36			250	Filter		
		37			250	Filter		
	B	39	162	108	50	Pellet		
		40			250	Pellet		
		41			250	Filter		
		42			250	Filter		
	C	44	177	113	50	Pellet		
		45			250	Pellet		
		46			50	Filter		
	**Extraction kit**							
01-20-2022	A	48	143	137	200	Pellet	3,400	PowerLyzer
		49				Pellet		PowerSoil Pro
		50				Filter		PowerSoil Pro
		51				Filter		PowerSoil Pro
	B	52	132	98		Pellet		PowerLyzer
		53				Pellet		PowerSoil Pro
		54				Filter		PowerSoil Pro
		55				Filter		PowerLyzer
	C	56	193	143		Pellet		PowerLyzer
		57				Pellet		PowerSoil Pro
		58				Filter		PowerSoil Pro
		59				Filter		PowerSoil Pro
	**Centrifugation speed**							
03-17-2022	A	74	508	447	200	Pellet	3,400	PowerSoil Pro
		75					15,000	
	B	79	132	110			3,400	
		80					15,000	
	C	82	210	157			3,400	
		84					15,000	
	**Filter vs pellets**							
04-21-2022	A	87	41	37	200	Filter	15,000	PowerSoil Pro
		88				Pellet+Filter		
		89				Pellet		
	B	90	76	50		Filter		
		91				Pellet+Filter		
		92				Pellet		
	C	94	100	57		Filter		
		95				Pellet+Filter		
		96				Pellet		

#### Sample volume and concentration

To determine the ideal sample volume to maximize microbial recovery, 50 and 250 mL aliquots from the same WWTPs were processed (Table 1).

The effect of centrifugation speed on sample concentration was assessed by processing samples at two speeds: a baseline of 3,400 × *g*, as per N’Guessan et al. (4), and an increased speed of 15,000 × *g*, following the protocol of Hendriksen et al. (2). Given the capacity limitations of the centrifuge bottles used in the 15,000 × *g* concentration step, a working volume of 200 mL was adopted for subsequent analyses. Pellets obtained from 200 mL influent samples were systematically split into two equal aliquots to ensure that the total mass recovered did not exceed 250 mg, the extraction limit recommended by the manufacturer. For these cases, the reference volume used for calculations was adjusted to 100 mL to maintain consistency in yield estimations.

We scrutinized the impact of using either pellets, filtered supernatants, or a combination of both. Our data set consisted of samples tested in volume and extraction experiments (Table 1) and 32 additional 200 mL influent and effluent wastewater samples collected during the summer of 2022 (see File S11 at http://bit.ly/4nNZioR).

After centrifugation, the pellets were recovered, and the supernatants were filtered on sterile 0.45 µm (Table 1) and 0.22 µm (File S11) mixed ester cellulose membranes (Thermo Scientific, US). The filters were inserted in 5 mL tubes with the filtration side facing inward to maximize the surface area available, and both pellets and filters were stored at −80°C until subsequent DNA extraction.

#### DNA extraction and quantification

DNA extraction was conducted using a DNeasy PowerLyzer Kit and DNeasy PowerSoil Pro Kit (Qiagen, DE) following the manufacturer’s protocols. A DNA extraction blank control was processed in parallel with the samples to monitor background DNA contamination.

Bead-beating conditions followed the specifications for each kit, with the PowerSoil Pro kit using a vortex adapter and the PowerLyzer kit requiring a more intensive bead-beating step. Due to the unavailability of the specified PowerLyzer homogenizer, we used the FastPrep-24 system as an alternative. The two kits were compared across various matrices at a consistent centrifugation speed (Table 1). DNA was eluted to a final volume of 100 µL.

DNA purity and concentration were assessed using an Invitrogen Qubit 4 fluorometer and a NanoDrop One spectrophotometer (Thermo Scientific, US), following the manufacturer’s instructions. We employed the dsDNA high-sensitivity (HS) mode on Qubit, which can detect nucleic acid concentrations as low as 0.005 to 120 ng/μL. For NanoDrop One, we used its dsDNA HS mode, which has a detection range from 2 to 27,500 ng/μL.

To evaluate the performance of these quantification methods, a total of 100 DNA samples extracted from wastewater (*n* = 81), sludge (*n* = 6), surface water (*n* = 10), and drinking water samples (*n* = 3) collected from 2020 to 2022 were used (see File S17 at http://bit.ly/4m4ycIp).

### Molecular analysis

#### 16S rRNA gene sequencing and analysis

The V4 region of the 16S rRNA gene was amplified using the modified Caporaso primers 515F and 806R (33, 34). The initial PCR protocol began with a denaturation step at 94°C for 3 min; 34 cycles of denaturation at 94°C for 45 s, annealing at 50°C for 60 s, and extension at 68°C for 90 s; and a final extension at 68°C for 10 min. A second round of PCR for barcoding attachment used a consistent forward primer and a variable reverse primer, assigning a unique identifier to each amplicon. This step included initial denaturation at 94°C for 3 min; 15 cycles of 94°C for 30 s, 59°C for 20 s, and 68°C for 45 s; and a final extension at 68°C for 5 min. Finally, PCR products from all the samples were pooled in equal proportions (50 ng) and sequenced on the Illumina MiSeq PE250 platform at Genome Québec (Canada).

#### Quantitative PCR for detecting ARGs

Prediluted samples (10 ng/µL) were analyzed for specific ARGs using the SmartChip qPCR system at Resistomap Oy, Finland. The selected ARGs (*bla*_TEM_, *bla*_SHV_, *bla*_OXA_, *bla*_CTX-M_, *bla*_KPC_, *bla*_NDM_, *bla*_IMP_, *qnr*A, *qnr*B, *mph*E, and *mef*A) encode resistance to β-lactams, macrolides, and fluoroquinolones, which represent some of the most used antimicrobial classes in the Canadian context (35). Their selection was informed by previous reports of detection in local wastewater samples and by their recognized clinical relevance and frequent association with mobile genetic elements, as described in studies such as Hendriksen et al. (2) and Zhang et al. (36). The 16S rRNA gene was used as a normalization reference, with DNA quantification targeting the 16S rRNA gene rather than its transcript.

The qPCR assays were performed using a SYBR-based approach without probes. The primer sequences used in this study were primarily designed and validated by Stedtfeld et al. (37), with the AY621 assay (mphE) obtained from Conrad et al. (38). The full list of primers is provided in Table S1.

Melting curve analysis and PCR efficiency checks were also conducted for each gene, setting a threshold cycle (Ct) of 27 as the detection limit (39–42). This Ct 27 threshold was selected based on the analytical pipeline used by Resistomap, which follows assay detection comparisons from Stedtfeld et al. (37). Their study evaluated different Ct thresholds and determined that Ct 27 provides an optimal balance between sensitivity and specificity, ensuring signals remain above background noise while minimizing the inclusion of unreliable low-abundance signals.

False positives, indicated by unspecific melting curves or multiple peaks, were excluded. The relative abundance of each ARG was calculated using the mean Ct from three replicates and the 2−ΔCt method (42), comparing the detected genes to the 16S rRNA gene.

### Data analysis

Sequencing data were processed using R software version 4.0.1. The DADA2 pipeline facilitated primer removal (Cutadapt v. 2.10), error correction, denoising, and taxonomic assignment using the Silva database (43). Operational taxonomic unit (OTU) clustering was performed at a 97% identity threshold. The phyloseq package version 1.34.0 was used to integrate the sequencing results with sample metadata for comprehensive analysis of microbial communities using alpha and beta diversity metrics (44).

Alpha analyses focused on the Chao1 and Shannon indices, calculated using the estimate_richness function. For beta diversity analysis, pairwise dissimilarities between microbial communities were calculated using principal coordinate analysis (ordinate) with Bray‒Curtis and Jaccard dissimilarity indices (distance) to discern distinctions in microbial communities from samples subjected to different processing conditions. Diversity plots were constructed using the ggplot2 package to visualize the relative abundance of the 10 most abundant genera within the samples, aggregating the remaining taxa into an “Other” category for streamlined visualization.

For ARG prevalence analysis, each researched gene’s average (relative to the 16S rRNA gene) was compared across different sample processing conditions. For filters versus pellets, a larger data set allowed for the estimation of the statistical significance of the differences observed. Data normality was first assessed using the Shapiro‒Wilk test (shapiro.test). For normally distributed data with equal variances (verified by Levene’s test using the (car::leveneTest), a t test (t.test) was applied; otherwise, a nonparametric Mann–Whitney U test (wilcox.test) was used to compare means between groups. Statistical significance was defined as *P* < 0.05. Charts were created using the Resistomap Dashboard (https://app.resistomap.com).

The concordance between the NanoDrop One and Invitrogen Qubit 4 measurements, as well as their alignment with the qPCR results, was calculated using Pearson and Spearman correlation coefficients. Samples below quantification limits were excluded, and those exceeding detection ranges were diluted and normalized. Scatter plots with regression lines visually display the correlations between the Invitrogen Qubit 4 and NanoDrop One measurements. Additionally, plots illustrating the correlations among the three quantification methods were created using Microsoft Excel 2010.

## RESULTS

In this study, we improved a wastewater sampling processing protocol using 38 subsamples, each characterized by a unique set of conditions. These subsamples were derived from 12 samples collected across three WWTPs over four consecutive months. The results cannot be compared across experiments because the initial samples differ between them. Table 2 outlines the DNA extraction yields and alpha diversity measures across the tested conditions.

**TABLE 2:** Alpha-diversity metrics across different processing conditions^*a*^

WWTP	Sample ID	Sample description	DNA concentration (Qubit)	(A260/A280)	(A260/A230)	Chao1	Shannon
A	34	Pellet 50 mL	23.6	1.72	1.55	612	4.64
	35	Pellet 125 mL	23.8	1.79	1.58	536	4.49
	36	Filter 250 mL	10.8	1.79	1.58	497	4.67
	37	Filter 250 mL	13.1	1.86	0.26	478	4.69
B	39	Pellet 50 mL	12	1.87	1.68	432	3.69
	40	Pellet 125 mL	19.8	1.82	1.56	334	3.41
	41	Filter 250 mL	13.4	1.85	1.67	507	4.72
	42	Filter 250 mL	8.7	1.87	0.29	479	4.74
C	44	Pellet 50 mL	27.8	1.83	1.27	418	3.76
	45	Filter 250 mL	11.4	1.82	1.47	431	4.28
	46	Filter 50 mL	64	1.84	0.3	586	4.94
A	48	Pellet PL	6.5	1.76	0.96	533	3.76
	49	Pellet PWP	12.1	1.82	0.28	484	3.7
	50	Filter PWP	2.59	1.84	0.19	232	3.46
	51	Filter PL	20.8	1.82	0.2	497	3.91
B	52	Pellet PL	13.8	1.81	1.32	333	2.63
	53	Pellet PWP	15.1	1.82	1.27	218	1.63
	54	Filter PWP	12.5	1.84	0.2	453	3.86
	55	Filter PL	19.3	–	–	218	2.32
C	56	Pellet PL	13.2	1.79	1.41	346	3.27
	57	Pellet PWP	33.2	1.86	0.71	366	3
	58	Filter PWP	Too low	–	–	346	3.13
	59	Filter PWP	8.5	–	–	434	4.13
A	74	Pellet 3,400 × *g*	18.2	1.71	0.62	332	3.26
	75	Pellet 15,000 × *g*	41.5	1.85	0.77	159	2.07
B	79	Pellet 3,400 × *g*	41.7	1.74	0.31	270	1.82
	80	Pellet 15,000 × *g*	51	1.82	1.07	201	1.6
C	82	Pellet 3,400 × *g*	12.8	1.87	0.53	225	2.34
	84	Pellet 15,000 × *g*	25.6	1.79	0.84	169	1.25
A	87	Filter	22.9	1.82	1.35	544	4.59
	88	Pellet+Filter	18.3	1.83	0.61	389	4.46
	89	Pellet	30.4	1.78	0.67	125	3.75
B	90	Filter	36.4	1.82	1.52	482	4.55
	91	Pellet+Filter	0.55	1.51	0.05	490	4.54
	92	Pellet	44.8	1.84	0.66	519	4.6
C	94	Filter	14.3	–	–	454	4.15
	95	Pellet+Filter	5.5	1.72	0.46	460	4.32
	96	Pellet	20.2		–	300	4.07

^*a*^ The DNA concentrations (ng/μL) measured with an Invitrogen Qubit 4, quality ratios measured with NanoDrop, and alpha diversity indices (Chao1 and Shannon) for samples processed using different methods. Notably, pellets obtained from initial volumes of 250 or 200 mL were divided after centrifugation into two aliquots of equal mass, thus representing equivalent original volumes of 125 and 120 mL, respectively. PL stands for DNeasy PowerLyzer PowerSoil Kit, and PWP for DNeasy PowerSoil Pro Kit. “–” Represents samples that could not be quantified using NanoDrop (insufficient volume).

### DNA recovery

Larger initial sample volumes and higher centrifugation speeds were generally associated with increased DNA recovery (Table 2). Direct comparisons between the filters and pellets showed that pellets usually yielded greater amounts of DNA, containing, on average, approximately 1.32 times more DNA per milliliter of the original sample than the filters. Regarding DNA extraction efficiency, the DNeasy PowerSoil Pro Kit slightly outperformed the DNeasy PowerLyzer Kit in recovering higher concentrations for 5 of the 11 ARGs from pellet samples, while the PowerLyzer recovered higher concentrations for 4 of them (Table 4). Both kits appear to be equivalent in terms of total DNA recovery (Table 2).

No significant correlation was observed between TSS or VSS and DNA recovery (R = 0.12, *P* = 0.51; Spearman’s ρ = −0.29, *P* = 0.117 for TSS; Pearson’s r = −0.09, *P* = 0.642; Spearman’s ρ = −0.30, *P* = 0.113 for VSS), suggesting that solid content had minimal impact on extraction efficiency. While the negative ρ values indicate a slight decreasing trend in DNA recovery with increasing TSS/VSS, these correlations were weak and not statistically significant (*P* > 0.05).

### Comparative analysis of DNA quantification techniques

**Fig 2:**
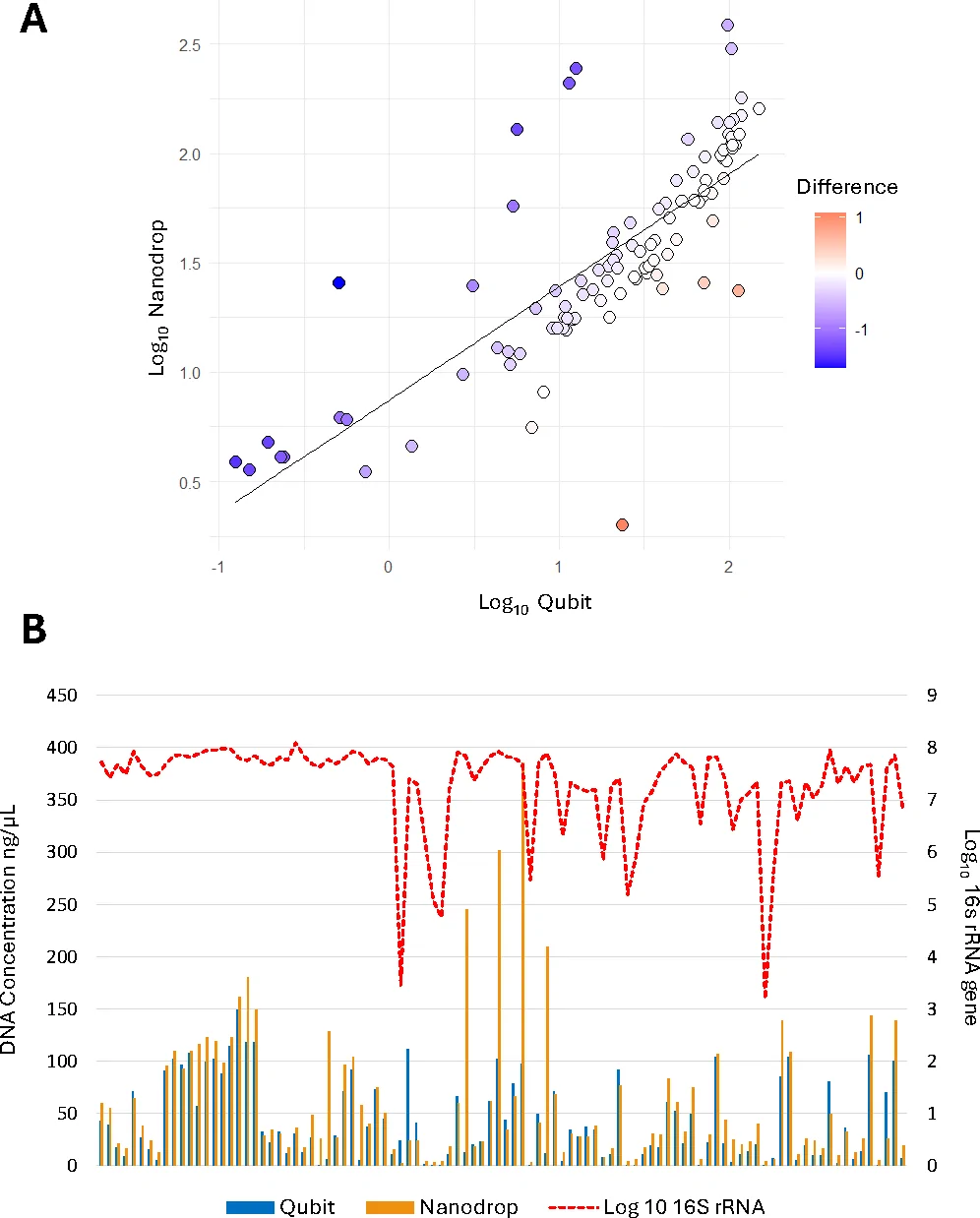
Correlation between the Invitrogen Qubit 4 and NanoDrop One DNA quantification. Panel A (scatter plot) shows the correlation between DNA concentrations determined by Invitrogen Qubit 4 fluorometry (x-axis) and NanoDrop One spectrophotometry (y-axis) on a logarithmic scale. Each point signifies the measurement from an individual sample. The color coding highlights the discrepancy between the measurements: blue indicates higher NanoDrop One values, red indicates higher Invitrogen Qubit 4 values, and lighter shades indicate closer agreement. The regression line drawn across the plot illustrates the general correlation trend between the two methods. The proximity of points to this regression line indicates the level of agreement between the Invitrogen Qubit 4 and NanoDrop One instruments, with points far from the line indicating greater divergence in the readings. Panel B is a dual-axis bar and line graph contrasting DNA concentration assessments obtained via Invitrogen Qubit 4 (blue bars) and NanoDrop One (orange bars) with the log-transformed 16S rRNA gene quantities (dotted red line) per sample. The left y-axis represents the DNA concentration in ng/µL, while the right y-axis indicates the log10-scaled 16S rRNA gene count. On the log scale, a single unit increase reflects a 10-fold increase in gene copy number, spanning from 10^1^ to 10^9^. Panel **A **refers to the scatter plot showing the correlation between Qubit and NanoDrop measurements, while Panel **B** refers to the dual-axis bar and line graph showing DNA concentrations and 16S rRNA gene quantities.

Our study revealed a significant positive correlation between the NanoDrop One and Invitrogen Qubit 4 DNA quantification readings, as illustrated by the scatter plot in Fig. 2, panel A. The figure shows a general alignment between the two methods, with white points indicating agreement. Nevertheless, in some instances, the DNA concentration readings were higher in one method than the other.

We employed both the Pearson correlation coefficient (R) and the Spearman correlation coefficient (ρ) to assess the linear and monotonic relationships between these methods and qPCR, with the latter considered the gold standard. The normality of the NanoDrop One data distributions, as indicated by Shapiro‒Wilk’s test and histogram analysis, and the non-normality of the Invitrogen Qubit 4 data (W = 0.86, *P* < 0.01) justify the use of these two distinct correlation tests. Values close to 1 or −1 indicate a strong positive or negative correlation, respectively, implying a consistent directional relationship between the compared variables.

The Pearson R-value of 0.78 (*P* < 0.01) for NanoDrop One versus Invitrogen Qubit 4 suggested a robust correlation, which was further substantiated by a Spearman ρ of 0.81 (*P* < 0.01), indicating a strong monotonic association. Conversely, a ρ of 0.66 (*P* < 0.01) for the correlation between Invitrogen Qubit 4 and qPCR denotes a reliable yet slightly weaker linear relationship. A Pearson R of 0.77 (*P* < 0.01) indicated a close alignment of readings provided by NanoDrop One with our qPCR results. Figure 2 in panel B shows the results of this analysis by contrasting DNA concentration measurements from both the Invitrogen Qubit 4 and NanoDrop One instruments with log-transformed 16S rRNA gene quantities.

To assess the microbial fraction of total DNA extracted, we compared 16S rRNA gene copy numbers with DNA concentrations measured by Qubit and Nanodrop. A moderate correlation was observed between 16S rRNA and total DNA, with slightly stronger association for Qubit (r = 0.63) than for Nanodrop (r = 0.54). These results suggest that while both methods capture general trends in biomass, Qubit quantification may better reflect the microbial component of complex samples, possibly due to its higher specificity for double-stranded DNA. The observed variability may partially reflect the presence of non-microbial DNA in untreated wastewater, such as host-derived or extracellular nucleic acids, and underscores the limitations of relying on total DNA concentration alone when estimating microbial load.

### Microbial diversity analysis

We analyzed the taxonomic composition and abundance of microbial communities in wastewater samples using barcoded sequencing of the 16S rRNA gene. The mean number of quality-filtered, nonchimeric reads per sample assigned to OTUs was approximately 100,000, indicating a robust sequencing depth. The number of reads per sample ranged from 14,635 to 122,664. In total, 7,194 unique OTUs were identified and further classified into 683 distinct genera.

Alpha (Chao1 and Shannon) and beta-diversity indices (Bray‒Curtis and Jaccard) were calculated for wastewater samples from different treatment plants and their aliquots processed under varying conditions (Tables 2 and 3).

The Chao1 index assesses species richness, reflecting the total number of distinct species within a community. The Shannon index measures the abundance and evenness of species distributions, providing an overall sense of diversity within a sample. The Bray‒Curtis index considers species abundance, while the Jaccard index is influenced by the presence or absence of species, emphasizing the contribution of rarer species.

**TABLE 3:** Comparison of beta-diversity metrics across different processing conditions^*a*^

WWTP	Sample pair	Bray‒Curtis	Jaccard
	Volume (12-07-2021)		
A	Pellet 50 mL–Pellet 125 mL	0.25	0.40
	Pellet 50 mL–Filter 50 mL	0.45	0.62
	Pellet 125 mL–Filter 250 mL	0.40	0.57
	Filter 50 mL–Filter 250 mL	0.17	0.29
B	Pellet 50 mL–Pellet 125 mL	0.12	0.22
	Pellet 50 mL–Filter 50 mL	0.54	0.71
	Pellet 125 mL–Filter 250 mL	0.55	0.71
	Filter 50 mL–Filter 250 mL	0.14	0.25
C	Pellet 50 mL–Filter 125 mL	0.36	0.53
	Pellet 50 mL–Filter 50 mL	0.57	0.73
	Filter 125 mL–Filter 50 mL	0.35	0.52
	Extraction Kit (01-20-2022)		
A	Pellet PL–Pellet PWP	0.15	0.26
	Pellet PL–Filter PL	0.19	0.31
	Filter PWP–Filter PL	0.59	0.74
	Filter PWP–Pellet PWP	0.61	0.76
B	Pellet PL–Pellet PWP	0.24	0.38
	Pellet PL–Filter PL	0.32	0.49
	Filter PWP–Filter PL	0.32	0.49
	Filter PWP–Pellet PWP	0.47	0.64
C	Pellet PL–Pellet PWP	0.19	0.32
	Filter PWP–Pellet PWP	0.15	0.27
	Filter PWP–Pellet PL	0.15	0.25
	Centrifugation speed (02-17-2022)		
A	Pellet 3,400 × *g*–Pellet 15,000 × *g*	0.28	0.44
B	Pellet 3,400 × *g*–Pellet 15,000 × *g*	0.12	0.21
C	Pellet 3,400 × *g*–Pellet 15,000 × *g*	0.22	0.21
	Filter vs pellets (04-21-2022)		
A	Filter–Pellet	0.76	0.86
	Filter–Pellet+Filter	0.37	0.54
	Pellet–Pellet+Filter	0.64	0.78
B	Filter–Pellet	0.25	0.40
	Filter–Pellet+Filter	0.36	0.53
	Pellet–Pellet+Filter	0.35	0.51
C	Filter–Pellet	0.29	0.45
	Filter–Pellet+Filter	0.18	0.31
	Pellet–Pellet+Filter	0.29	0.45

^*a*^ Beta diversity was assessed through pairwise comparisons using Bray‒Curtis and Jaccard dissimilarity metrics. PL stands for DNeasy PowerLyzer PowerSoil Kit, and PWP for DNeasy PowerSoil Pro Kit.

Regarding volume, the alpha indices were relatively stable within each WWTP (Table 2). While some variations were observed among the plants, these differences were not significant. The mean Chao1 indices for plants A, B, and C were 530.75 (±59.30), 438.00 (±75.93), and 478.33 (±93.47), respectively. The Shannon indices followed a similar pattern, with values of 4.62 (±0.09), 4.14 (±0.69), and 4.33 (±0.59) for the respective plants.

**Fig 3:**
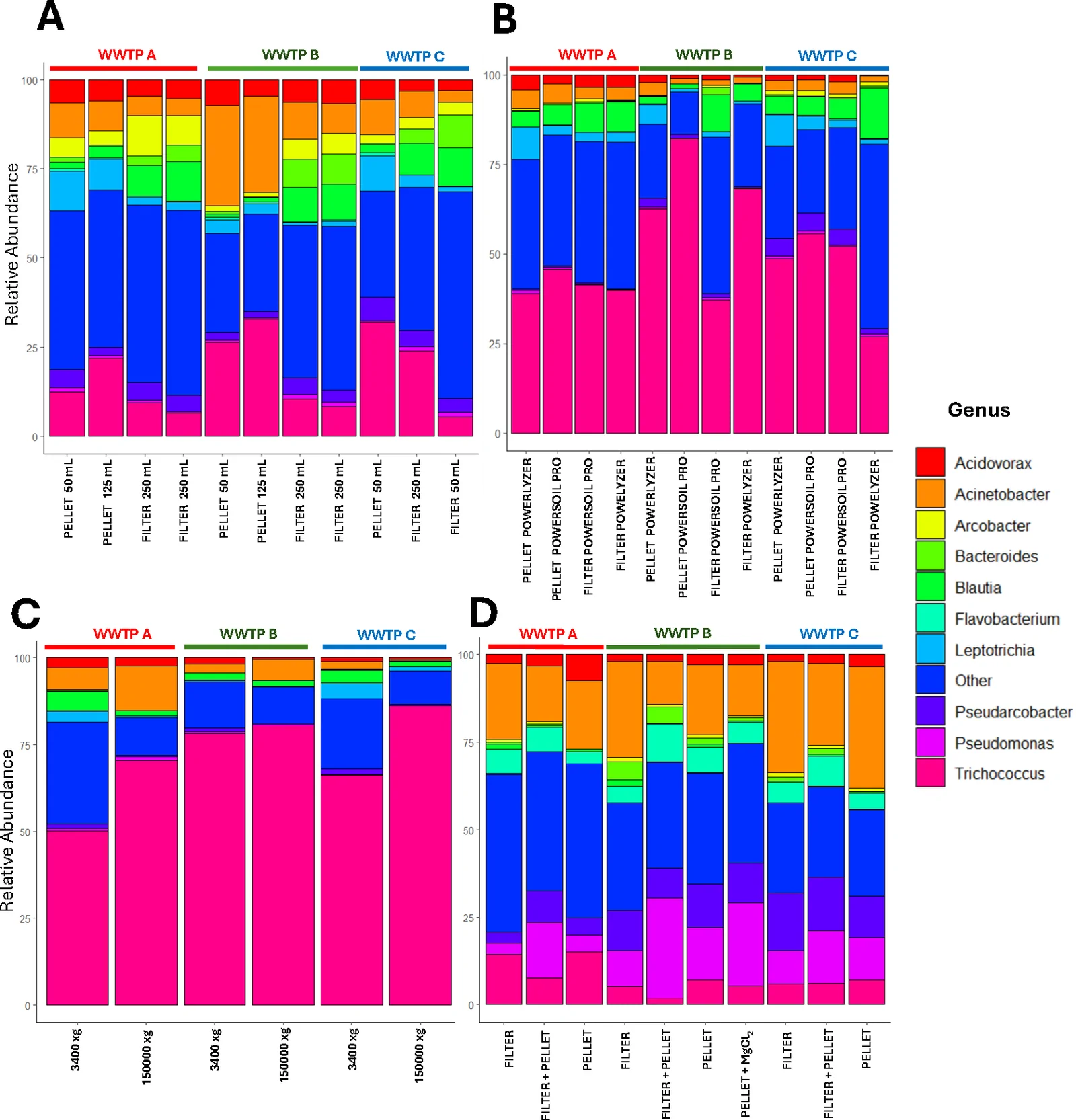
Bacterial community composition across different processing conditions. Panels **A–D of Fig. 3** display stacked bar charts representing the bacterial community composition across various wastewater samples from three treatment plants (WWTP** A, B, and C**). The color segments of each bar denote the relative abundance of the ten most prevalent bacterial genera, with the color key indicating the genus identification. Panel A shows the microbial profiles of the pellet and filter samples processed from 50 and 250 mL volumes. Panel B compares the bacterial diversity in samples extracted using PowerSoil Pro and PowerLyzer kits. Panel C focuses on the community structure after centrifugation at 3,400 × *g* and 15,000 × *g*. Finally, panel D highlights differences in bacterial abundance when using filters, pellets, or a combination of both, including a condition with and without MgCl_2_.

The analysis of aliquots with different volumes from the same treatment facility revealed Bray‒Curtis values ranging from 0.12 to 0.57 and Jaccard values ranging from 0.22 to 0.73, reflecting a spectrum of dissimilarity. Notably, 50 mL aliquots consistently demonstrated lower dissimilarity. Conversely, aliquots of 125 and 250 mL exhibited greater dissimilarity, suggesting that larger sample sizes capture a more diverse array of species, including less common species.Fig. 3 , panel A, provides a clear visual representation of these findings, illustrating the relative abundance of microbial genera in aliquots of varying volumes.

By examining the impact of centrifugation speed on microbial community composition, we noted a discernible effect on alpha diversity. While there was a trend toward lower richness at higher centrifugation speeds (Table 2), this trend does not imply a significant loss of ecological value or sample representativeness. The modest dissimilarity values—especially as indicated by Bray‒Curtis indices of 0.28, 0.12, and 0.22—denote that despite changes in alpha diversity, the overall composition of the community is maintained across different centrifugation speeds, with no drastic shifts in the relative abundance of species. This phenomenon is graphically depicted in Figure 3, Panel C, where similarities in community profiles at different centrifugation speeds can be observed.

**Fig 4:**
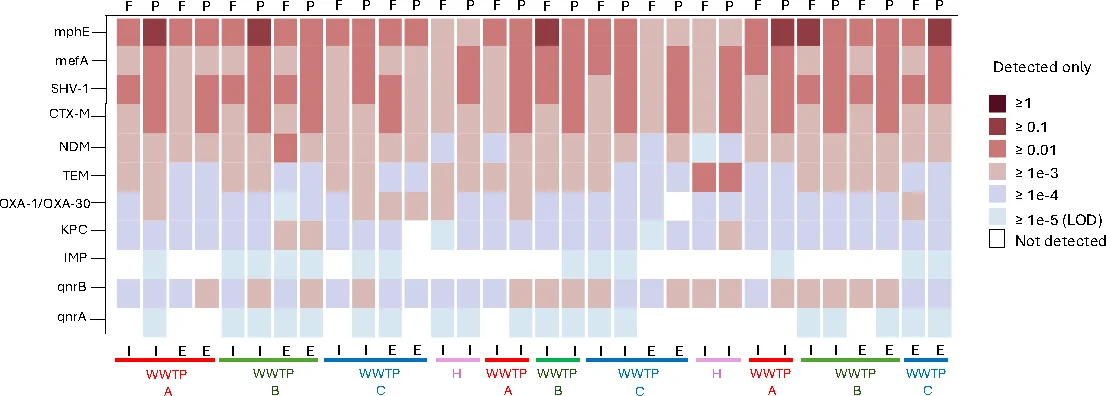
Heatmap of ARG detection in filter and pellet samples. The heatmap illustrates the relative abundance of selected ARGs in influent (I) and effluent (E) wastewater samples from three WWTPs (A, B, and C) and one Hospital (H). Each row represents a different ARG, while columns represent samples processed either as pellets (P) or through a 0.22 µm filter (F). The color intensity indicates the ARG abundance, with darker shading indicating higher levels. The absence of color denotes non-detection. The scale bar interprets detection levels, from the limit of detection (LOD) at ≥1e-5 to a maximum of ≥1. Samples collected on the same date from the same source were grouped together, allowing direct comparisons between pellet and filter processing methods within the same collection context.

The use of pellets in this study enabled the detection of a broad range of microbial taxa. However, when compared with filters processed under similar volume and extraction kit conditions, filters generally exhibited slightly higher richness and diversity (Tables 2 and 3). These findings highlight the importance of matrix-specific characteristics and support the need for methodological comparisons when interpreting microbial diversity data.

A similar tendency was observed in the experimental setup designed to investigate the effects of using filters, pellets, and their combination. The filters displayed broader species ranges, as indicated by an average Chao1 of 493 (±46.06) and a Shannon index of 4.43 (±0.14). However, the pellet method still effectively captured substantial diversity, with an average Chao1 of 315 (±197.41) and a Shannon index of 4.14 (±0.43). Notable differences in community composition between the pellet and filter methods were observed in WWTP A, but these differences were less pronounced in WWTPs B and C (Table 2 and Fig. 4, panel D).

The comparative analysis of extraction kits within the same WWTP samples showed that the essential community composition remained relatively consistent across both extraction methods, as evidenced by beta diversity comparisons.

In WWTP A, the PowerLyzer kit yielded a mean Bray‒Curtis index of 0.17 (±0.02) and a Jaccard index of 0.285 (±0.025), while the PowerSoil Pro kit produced higher values with a Bray‒Curtis index of 0.60 (±0.01) and a Jaccard index of 0.750 (±0.01), suggesting some variability in filter comparisons. In WWTP B, the PowerLyzer kit showed a mean Bray‒Curtis index of 0.28 (±0.04) and a Jaccard index of 0.435 (±0.055), whereas the PowerSoil Pro kit yielded a Bray‒Curtis index of 0.395 (±0.075) and a Jaccard index of 0.565 (±0.075), indicating moderate differences. Results for WWTP C followed the same trend, showing consistent values between methods, with the PowerLyzer kit yielding a Bray‒Curtis index of 0.17 (±0.02) and a Jaccard index of 0.285 (±0.035), indicating minimal variation.

These findings suggest that, although some variability exists depending on the kit used, the overall microbial community structure remains similar and comparable across both extraction methods. These observations are visually substantiated by Fig. 4, panel B.

### Comparative analysis of ARGs under different test conditions

All primers used in this study had qPCR efficiency values within the accepted range for qPCR assays (1.70–2.10), minimizing potential bias. While a few individual reactions for *qnr*A, *bla*_SHV_, and *bla*_OXA-1/30_ had efficiency values slightly below 1.70, their mean efficiency remained within the expected range (1.86–1.97). The full efficiency data, including efficiency ranges and mean values for each assay, are presented in Table S1.

Our research assessed the prevalence of specific ARGs and the 16S rRNA gene using quantitative PCR. The mefA gene exhibited the highest abundance, with a mean of 1.1E-01 (0.11), while *bla*_IMP-1_ was the least abundant, with a mean of 3.0E-05 (0.00003). Table 4 delineates the variability in the relative abundance of ARGs under different experimental conditions.

**TABLE 4:** Relative abundance of resistance genes compared across different testing conditions (higher means are indicated in bold)^*a*^

Gene	Mean of relative abundance (to the 16S rRNA gene)						*P* value filter vs pellet
	3,400 ×*g*	15,000 × *g*	PowerSoil Pro	PowerLyzer	Filter	Pellet	
mefA	3.0E-02	**3.7E-02**	**1.1E-01**	6.6E-02	7.7E-03	**2.7E-02**	**<0.01**
mphE	2.8E-02	**5.0E-02**	**2.3E-02**	1.1E-02	4.3E-02	**4.8E-02**	0.60
*bla*_SHV_1_	2.1E-02	**2.6E-02**	3.0E-02	3.0E-02	1.0E-02	**2.2E-02**	**<0.01**
*bla*_CTX- M_	7.8E-03	**9.9E-03**	1.0E-02	1.1E-02	5.8E-03	**1.1E-02**	**<0.01**
*bla*_NDM_	2.1E-03	**3.9E-03**	**2.0E-03**	1.5E-03	2.4E-03	**3.4E-03**	**<0.05**
*bla*_TEM_	4.7E-04	**5.4E-04**	5.5E-04	**1.4E-03**	2.5E-03	**2.6E-03**	0.53
*bla*_OXA1_/*bla*_OXA30_	6.3E-04	**1.3E-03**	**2.9E-04**	2.1E-04	6.0E-04	**7.0E-04**	0.26
*bla*_KPC_	3.9E-04	**6.1E-04**	**7.1E-04**	5.8E-04	4.0E-04	**6.0E-04**	0.07
*bla*_IMP_1_	3.0E-05	**3.4E-05**	1.1E-05	**2.2E-05**	1.0E-05	1.0E-05	0.80
qnrB	7.2E-04	**9.6E-04**	6.0E-04	**7.2E-04**	1.0E-03	**1.9E-03**	**<0.01**
qnrA	2.4E-05	**6.8E-05**	3.4E-05	**4.0E-05**	2.0E-05	**3.0E-05**	0.17

^*a*^ A comparative analysis of the mean relative abundance of various ARGs normalized to the 16S rRNA gene. Paired data points from identical samples were subjected to different conditions, ensuring a valid comparison of the influence of each experimental variable on ARG abundance. For centrifugation speeds and DNA extraction kits, the dataset was limited to those used in preliminary tests, hence representing a smaller sample size. Conversely, the filter versus pellet comparison drew upon a more extensive dataset that allowed for robust statistical evaluation, with significant differences highlighted in bold. Statistical significance was determined using the t test or the Wilcoxon rank-sum test based on the results of Shapiro‒Wilk normality tests and Levene's test for equality of variances. The notation “xE-y” in scientific format succinctly represents smaller magnitudes, facilitating their interpretation. For instance, a value of 3.0E-02 translates to 3.0 × 10^−2 ^or 0.03 in standard decimal notation, while 3.0E-01 equates to 3.0 × 10^−1 ^or 0.3, rendering the latter nearly an order of magnitude greater than the former.

Centrifugation at 15,000 × *g* resulted in greater relative abundances than centrifugation at 3,400 × *g*, suggesting a correlation between increased centrifugal force and ARG recovery. The ARG abundance determined from the different DNA extraction kits varied, with no evident consistent trends. For instance, the DNeasy PowerSoil Pro Kit yielded higher means for the mefA gene, while the DNeasy PowerLyzer Kit was associated with higher *mph*E abundances. However, the limited number of observations for these comparisons restricts our ability to draw conclusions.

In contrast, the comparison between filters and pellets was supported by a more extensive data set, enabling robust statistical analysis. The results indicated that ARGs, such as mefA, *bla*_SHV-1_, *bla*_CTX-M_, and *qnr*B, were significantly more abundant in the pellet samples. The heatmap in Fig. 4 underlines these trends, showing that pellets (*n* = 16) frequently harbor ARGs at higher concentrations than filters.

Due to the limited availability of residual DNA from the preliminary tests, we could not explore the impact of sample volume variations on ARG abundance.

## DISCUSSION

In this investigation, we assessed the impact of different processing methods on wastewater samples, aiming to establish an improved protocol for AMR monitoring studies.

In our study, analyzing larger aliquots led to increased DNA recovery, yet this did not necessarily translate to greater microbial diversity. Previous research, such as that of Zhou et al. (21), suggested that larger initial sample volumes generally yield higher detection rates for low-concentration targets, such as *Salmonella typhi*, offering a broader perspective of the microbial community. Nonetheless, the results can vary. For example, Huijbers et al. (17) observed no direct correlation between the highest or lowest volume of wastewater and the diversity or concentration of *Escherichia coli* in composite samples.

The alpha diversity indices remained relatively consistent across each WWTP. This result might be attributed to the saturation of the binding capacity of the DNA extraction kit used. Although the DNeasy PowerLyzer Kit is designed to remove inhibitory substances from environmental samples (45), the concentration of inhibitors in larger water aliquots may exceed the kit’s capacity for effective removal, particularly in sewage samples (46–48). This could result in selective DNA isolation, potentially skewing microbial diversity despite higher total DNA yields. Moreover, the presence of these inhibitors might adversely affect downstream processes, such as 16S rRNA sequencing, leading to an underrepresentation of certain microbial components (15).

Despite technical constraints preventing a full evaluation of the effects of different volumes on the prevalence and abundance of ARGs, using 200 mL volumes was effective at ensuring ARG recovery, as evidenced by most of the target analytes being detected in the tested samples, with prevalence rates ranging from 100% to 54.3% (Table 3).

We compared two distinct centrifugation protocols—3,400 × *g* and 15,000 × *g* for 15 min—using the DNeasy PowerSoil Pro Kit. While these speeds have been used in previous studies (2, 4), their direct impact on DNA recovery has not been previously established. Our findings indicate a trend toward lower microbial richness at higher centrifugation speeds. However, despite the observed changes in alpha diversity, the overall composition of the microbial community was largely maintained across the different speeds, with no significant shifts in the relative abundance of the species. Moreover, higher speeds were found to be associated with enhanced ARG recovery, a key focus of our study. This observation likely reflects the improved pelleting efficiency of smaller particles, such as extracellular DNA or cell debris, which are essential for ARG detection. Similar approaches have been reported in studies (49) focusing on ultracentrifugation for viral recovery in large volumes, where higher speeds significantly improve the recovery of smaller particles (e.g., viruses) from complex matrices. This evidence influenced our decision to use this centrifugation speed for further tests.

Initially, our preliminary tests employed 47 mm diameter filters with a pore size of 0.45 µm following an adapted version of Guessan et al.’s protocol for SARS-CoV-2. Their approach included the addition of magnesium chloride (MgCl_2_) to a final concentration of 25 mM, which aids in the retention of viruses when using larger pore diameters (4, 50). However, relying on size-based retention is generally more suitable for bacterial recovery (51, 52).

For bacterial capture, filter pore sizes between 0.1 and 0.45 µm are used, with 0.22 µm being the most common due to its greater efficiency in capturing a broader range of bacterial sizes, thus potentially offering a more comprehensive representation of the microbial community (15, 51). Considering the size and natural differences between viruses and bacteria, we later transitioned to using 0.22 µm filters.

Our findings revealed that filters typically display slightly greater richness and diversity, as reflected by the broader species range indicated by the Chao1 indices, with an average value of approximately 493.33 compared to 323.67 for pellets.

However, pellets also effectively capture a substantial amount of microbial variation, proving to be a practical alternative in microbial diversity studies, particularly in wastewater analysis (24). In such contexts, the use of filters can be laborious and time-consuming due to the highly concentrated nature of the samples (15). Moreover, our results suggest that while filters may provide a broader snapshot of microbial diversity, pellets are more efficient at recovering ARGs. This is evidenced by the notably higher abundance of ARGs, such as mefA, *bla*_SHV-1_, *bla*_CTX-M_, and qnrB, in the pellet samples. These findings are in line with the results of Liu et al. (53), who observed that centrifugation-based workflows outperformed the filtration-based ones in terms of genome copies for 16S rRNA gene and intI1 (*P* < 0.05).

DNA yields ranged from 8.5 to 51.0 ng/mL across samples with TSS and VSS concentrations spanning 41–508 mg/L and 37–447 mg/L, respectively. A moderate to strong positive association was observed between DNA yield and suspended solids (R = 0.64 for TSS and R = 0.72 for VSS), reflecting the intuitive effect of biomass content on the total amount of nucleic acids recovered. In contrast, weak correlations between diversity indices and suspended solids (R = 0.26 for TSS and R = 0.36 for VSS) indicate that higher biomass does not necessarily translate into increased microbial richness. These findings, along with divergent DNA yields and diversity levels in samples with comparable solids concentrations, underscore the role of protocol-specific variables in shaping both the quantity and representativeness of recovered nucleic acids, potentially enhancing analytical outcomes even in samples with lower biomass.

According to our comparative study of the DNeasy PowerSoil Pro and DNeasy PowerLyzer Kits, the PowerSoil Pro was more efficient at extracting DNA from pellet samples. This enhanced performance may be due to the distinct protocols and chemical compositions of the kits used (45, 54). Notably, the PowerLyzer kit required multiple incubation steps at 4°C, whereas the PowerSoil Pro did not. These additional steps could influence overall efficiency by affecting cell lysis or DNA recovery.

Both kits incorporate a bead-beating step, which is essential for cell disruption and bacterial desorption from sediments—particularly crucial in complex matrices, such as wastewater (47, 55). In our study, the PowerSoil Pro kit employed a vortex adapter for bead-beating, while the PowerLyzer kit required a more intensive homogenization step, performed using the FastPrep-24 system. Previous research (56) shows that the bead-beating step plays a crucial role, given its ability to lyse bacteria with particularly resistant cell walls, such as non-tuberculous mycobacteria, ensuring higher DNA yields and the recovery of a diverse microbial community.

However, the DNeasy PowerSoil Pro Kit employs a two-step washing process utilizing a wash buffer to remove proteins and other nonaqueous contaminants, followed by an ethanol-based wash to further purify DNA adhered to the silica filter membrane. On the other hand, the DNeasy PowerLyzer Kit uses two precipitation steps and a single wash step, which may result in less comprehensive DNA purification. Nonetheless, despite these protocol variations affecting DNA recovery, both kits effectively maintained the overall microbial community structure, as evidenced by consistent beta diversity comparisons.

In the existing literature, there are numerous comparisons between these two extraction protocols (27, 30), though fewer studies have focused on their performance for environmental samples, which often contain more complex matrices and varied concentrations of nucleic acids. Here, we evaluate the performance of both techniques on DNA extracted from various water samples, including not only wastewater influents but also effluents, surface water, and drinking water, to encompass a spectrum of sample concentrations.

The analysis of DNA quality ratios reveals that A260/280 values remained within acceptable ranges across all protocols, indicating effective removal of protein contaminants, while A260/230 ratios varied substantially, particularly in pellet-based samples. This suggests that biomass concentration may co-purify residual organic matter or salts, which could interfere with enzymatic downstream applications in some cases. When comparing extraction kits, PowerLyzer yielded higher A260/230 values (1.00 ± 0.50) than PowerSoil Pro (0.53 ± 0.47), suggesting more effective removal of such contaminants. Nonetheless, A260/280 values were comparable between kits (1.80  ± 0.03 vs 1.84 ± 0.02), supporting the overall integrity of the DNA extracts.

In our study, the strong Pearson R and Spearman ρ values for NanoDrop One versus Invitrogen Qubit 4 reflect a robust linear relationship and a strong monotonic trend. This finding suggested that as the DNA concentration reported by one method increases, so does the other’s, particularly at higher DNA concentrations. Furthermore, a tighter clustering of data points along the line of best fit at higher log concentrations in our scatter plot indicates stronger agreement between the two methods. This finding is consistent with those of Billington et al. (27), who suggested that NanoDrop One and Invitrogen Qubit 4 instruments perform similarly well when assessing higher DNA concentrations. Conversely, at lower DNA concentrations, there is a visible spread of data points away from the line, with more pronounced color differences indicating greater discrepancies between the methods.

These discrepancies may stem from inherent differences in the principles underlying each method. The Qubit 4 employs a fluorescent dye that specifically binds to double-stranded DNA, which might not interact uniformly with all DNA bases at very low concentrations. In contrast, the NanoDrop One relies on spectrophotometry, a technique that might be prone to interference from contaminants, such as proteins and salts, potentially skewing measurements at lower concentrations (57). These methodological nuances might explain the observed differences and warrant further investigation into their implications for low-concentration DNA assessments. While we found a strong correlation between NanoDrop One and qPCR data, suggesting close alignment, the correlation between the Invitrogen Qubit 4 and qPCR was slightly weaker.

### Strengths and limitations

This research represents a pioneering effort to examine the effects of sample processing procedures in wastewater for surveillance. Its comprehensive approach, investigating the impact of different sample volumes, centrifugation speeds, pellets versus filters, and DNA extraction methods, offers valuable insights into the complexities of microbial diversity and ARG prevalence in wastewater.

However, this study has its limitations since the preliminary tests involved a limited number of samples. Each tested parameter was assessed independently, with pairwise comparisons conducted within aliquots from the same wastewater treatment plant to minimize confounding effects. Nonetheless, future studies should incorporate a fully factorial design with replicates to enable robust statistical analyses.

Additionally, while differences in DNA yield between methods were observed, this study did not incorporate specific inhibition controls, such as spike-ins at the extraction step or total nucleic acid dilution experiments, to assess the impact of inhibitors on downstream analyses. Similarly, the absence of spike-ins of known microbial taxa limits the ability to evaluate potential biases introduced by DNA extraction methods, bead beating intensities, and primer choices, which may influence microbial diversity estimates. Future studies should integrate such controls to refine interpretations and enhance methodological robustness.

Acknowledging these limitations and considering the variable concentrations of microbial components in wastewater, the authors emphasize the need to conduct pretests in specific contexts. This approach is critical for determining the optimal sample volume for accurate and representative analysis. The concentration of microbial content in wastewater is strongly influenced by geographical location, seasonal changes, and other environmental conditions, necessitating localized and seasonal adaptability in protocol development.

Despite these limitations, this study provides foundational evidence that can be instrumental in establishing a framework for processing wastewater for AMR monitoring purposes. This research lays the groundwork for future studies focusing on protocol refinement in WS for AMR.

### Conclusion

In this study, we compared methods for concentrating wastewater for AMR research. The process involved concentrating 200 mL samples via centrifugation at 15,000 × *g* for 15 min, followed by DNA extraction from the pellets using the DNeasy PowerSoil Pro Kit (Qiagen, DE). While this approach still requires further validation in specific scenarios, it may serve as a guide for future work in environmental monitoring of ARGs.

## Data Availability

All datasets generated and analyzed during this study, along with the R scripts used for data processing, are openly accessible in a GitHub repository: http://bit.ly/3V9AQBz, ensuring the transparency and reproducibility of our findings. The repository includes raw data, processed datasets, metadata, and R scripts used for data processing, analysis, and visualization. Comprehensive descriptions of all files are provided in the repository’s README.md. The raw FASTQ files can be accessed through a OneDrive link provided in the associated GitHub repository, enabling further exploration of the sequencing data. For additional details or inquiries, please contact the corresponding author.

## SUPPLEMENTAL MATERIAL

Table S1 spectrum.02089-25-s0001.docx Primer sequences and qPCR efficiency values.

ASM does not own the copyrights to Supplemental Material that may be linked to, or accessed through, an article. The authors have granted ASM a non-exclusive, world-wide license to publish the Supplemental Material files. Please contact the corresponding author directly for reuse.
