# Supplementary material for: Comparative analysis of wastewater sample processing methods for antimicrobial resistance surveillance
Source: Microbiol Spectr. 2026 Jun 15;14(7):e02089-25. doi: 10.1128/spectrum.02089-25 (PMC13340250; doi:10.1128/spectrum.02089-25)
Supplement: Table S1 — Primer sequences and qPCR efficiency values. [file spectrum.02089-25-s0001.docx]

| Assay | Target | Forward primer | Reverse Primer | Efficiency (range) | Efficiency (mean) | Reference |
| --- | --- | --- | --- | --- | --- | --- |
| AY1 | 16S rRNA | CCTACGGGAGGCAGCAG | ATTACCGCGGCTGCTGGC | 1.73-2.17 | 1.94 | [Stedtfeld  *et al*. (2018)](https://academic.oup.com/femsec/article/94/9/fiy130/5057470) |
| AY432 | *bla*_CTX-M_ | GCGATAACGTGGCGATGAAT | GTCGAGACGGAACGTTTCGT | 1.79-1.97 | 1.89 |  |
| AY130 | *bla*_IMP_ | GGAATAGAGTGGCTTAATTC | GGTTTAACAAAACAACCACC | 1.72-1.99 | 1.88 |  |
| AY152 | *bla*_NDM_ | GGCCACACCAGTGACAATATCA | CAGGCAGCCACCAAAAGC | 1.75-1.94 | 1.88 |  |
| AY104 | *bla*_SHV_ | TTGACCGCTGGGAAACGG | TCCGGTCTTATCGGCGATAAAC | 1.67-2.04 | 1.86 |  |
| AY120 | *bla*_OXA-1/30_ | CGACCGAGTATGTACCTGCTTC | TCAAGTCCAATACGACGAGCTA | 1.64-2.05 | 1.97 |  |
| AY439 | *bla*_TEM_ | CGCCGCATACACTATTCTCAG | GCTTCATTCAGCTCCGGTTC | 1.71-1.99 | 1.90 |  |
| AY440 | *bla*_KPC_ | GCCGCCAATTTGTTGCTGAA | GCCGGTCGTGTTTCCCTTT | 1.7-1.95 | 1.87 |  |
| AY95 | *qnr*A | AGGATTTCTCACGCCAGGATT | CCGCTTTCAATGAAACTGCAA | 1.53-2.07 | 1.87 |  |
| AY96 | *qnr*B | CGACGTTCAGTGGTTCAGATCTC | GCCAAGCCGCTCCATGAG | 1.77-1.99 | 1.89 |  |
| AY538 | *mef*A | TAATTATCGCAGCAGCTGGTTC | GTTCCCAAACGGAGTATAAGAGTG | 1.76-2.14 | 1.95 |  |
| AY621 | *mph*E | TGGTATAAGTGAGCAATTGGAAACCCGCTA | TTGACCAATCAATAACGCCTGAAACAGCTC | 1.66-2.1 | 1.96 | [Conrad *et al*.  (2020)](https://www.frontiersin.org/journals/veterinary-science/articles/10.3389/fvets.2020.00208/full) |

**Table S1. Primer sequences and qPCR efficiency (range and mean) for 11 antimicrobial resistance genes (ARGs) and the 16S rRNA gene.**

This table presents the primer sequences and qPCR efficiency values for the 11 ARGs and the 16S rRNA gene quantified in this study. It includes the assay name (Assay), target gene (Target), forward and reverse primer sequences, observed efficiency (range and mean), and the reference for the original primer design and validation research (Stedtfeld *et al.*, 2018; Conrad *et al.,* 2020).
